# Ability of γδ T cells to modulate the Foxp3 T cell response is dependent on adenosine

**DOI:** 10.1371/journal.pone.0197189

**Published:** 2018-05-17

**Authors:** Dongchun Liang, Jeong-Im Woo, Hui Shao, Willi K. Born, Rebecca L. O'Brien, Henry J. Kaplan, Deming Sun

**Affiliations:** 1 Doheny Eye Institute and Department of Ophthalmology, David Geffen School of Medicine at UCLA, Los Angeles, CA, United States of America; 2 Department of Ophthalmology and Visual Sciences, Kentucky Lions Eye Center, University of Louisville, Louisville, KY, United States of America; 3 Department of Biomedical Research, National Jewish Health, Denver, CO, United States of America; Universite Paris-Sud, FRANCE

## Abstract

Whether γδ T cells inhibit or enhance the Foxp3 T cell response depends upon their activation status. The critical enhancing effector in the supernatant is adenosine. Activated γδ T cells express adenosine receptors at high levels, which enables them to deprive Foxp3^+^ T cells of adenosine, and to inhibit their expansion. Meanwhile, cell-free supernatants of γδ T cell cultures enhance Foxp3 T cell expansion. Thus, inhibition and enhancement by γδ T cells of Foxp3 T cell response are a reflection of the balance between adenosine production and absorption by γδ T cells. Non-activated γδ T cells produce adenosine but bind little, and thus enhance the Foxp3 T cell response. Activated γδ T cells express high density of adenosine receptors and have a greatly increased ability to bind adenosine. Extracellular adenosine metabolism and expression of adenosine receptor A2ARs by γδ T cells played a major role in the outcome of γδ and Foxp3 T cell interactions. A better understanding of the functional conversion of γδ T cells could lead to γδ T cell-targeted immunotherapies for related diseases.

## Introduction

Recent studies from several laboratories [1–5], including ours [6–9], have demonstrated that γδ T cells have a significant regulatory effect on autoimmune diseases [6–9]. The outcomes can be either enhancing [7,10,11] or inhibiting [12,13]. Our recent studies demonstrated that activated γδ T cells have an increased enhancing effect on the autoimmune response [7,14]; that the regulation of immune responses by γδ T cells and ATP/adenosine metabolism are intimately connected [15–18]; that competitive binding of adenosine among immune cells plays a key role in the outcome [15,18]. Clarifying the mechanism by which γδ T cells switch their regulatory influence should allow more effective manipulation of autoimmune responses.

Activated γδ T cells had an increased expression of high-affinity adenosine receptors (A2ARs) and decreased expression of CD73, which converts ATP/AMP into adenosine [19,20]. Whether such changes accounted for functional conversion had not been determined. Herein, we show that activated γδ T cells have an inhibitory effect on the Foxp3 T cell response. This inhibition relies on the expression of A2ARs at a higher density on γδ T cells; thus, these cells have a greater adenosine-binding ability than other immune cells, including αβ T cells and myeloid cells [15]. Preferential binding of adenosine by γδ T cells diminishes adenosine suppression of αβ T cells, leading to enhanced autoimmune responses. Our results demonstrate that activated γδ T cells enhance the autoimmune response, in part, because they inhibit the Foxp3 T cell response more effectively. Increased expression of A2ARs enables activated γδ T cells to remove adenosine effectively. In addition, binding of adenosine by γδ T cells also promotes γδ T cell activation [15,18]. We propose that a better understanding of the activation-dependent, adenosine-related functional conversion of γδ T cells could lead to γδ T cell-targeted immunotherapies in autoimmunity and other conditions affected by Foxp3^+^ regulatory T cells.

## Materials and methods

### Animals and reagents

All animal studies conformed to the Association for Research in Vision and Ophthalmology Statement on the Use of Animals in Ophthalmic and Vision Research. Institutional approval (Protocol number: ARC#2014-029-03A) was obtained from the Institutional Animal Care and Use Committee of the Doheny Eye Institute, University of California Los Angeles, and institutional guidelines regarding animal experimentation were followed. Veterinary care was provided by IACUC faculty. Immunized animal that displays swelling joints were either be humanely euthanatized or administered an analgesic (buprenorphine, 0.1 mg/kg sc. twice daily or ketoprofen, 2 mg/kg sc. daily) until the swelling resolves. By the end of the study, mice were euthanized by cervical dislocation after an injection of over dosed Ketamine and xylazine prior to tissue collection.

Female C57BL/6 (B6) TCR-δ^-/-^ mice on the B6 background were purchased from Jackson Laboratory (Bar Harbor, ME). A2AR^-/-^ mice were kindly provided by Dr. Jiang-Fen Chen of Boston University [21]. Animals were housed and maintained in the animal facilities of the University of California, Los Angeles (UCLA). FITC-, PE-, or allophycocyanin-conjugated Abs against mouse CD4, Foxp3, αβ T cell receptor (TCR), or γδ TCR and their isotype control Abs were purchased from Biolegend (San Diego, CA). The non-selective AR agonist 50-N-ethylcarboxamidoadenosine (NECA); selective A1R antagonist (DPCPX) [22–24]; selective A2AR antagonist (SCH58261) [25,26]; selective A2BR antagonist (MRS1754) [27]; and selective A3R antagonist (MRS 1220) [28] were purchased from R&D (Minneapolis, MN).

Recombinant murine IL-2 was purchased from R & D Systems (Minneapolis, MN). FITC- or PE-conjugated Foxp3 and isotype control antibodies were purchased from e-Bioscience (San Diego, CA). ADA was a gift from Sigma-Tau Pharmaceuticals Inc. (Gaithersburg, MD).

### T cell preparation

TCRαβ and CD3^+^ T cells were purified from B6 or TCR-δ^-/-^ mice immunized with the human interphotoreceptor retinoid-binding protein (IRBP) peptide IRBP_1-20_, as described previously [6,8,14]. Non-activated and activated γδ T cells were prepared by isolation from naïve and immunized mice, respectively [15,18,29]. Nylon wool-enriched splenic T cells from naïve or immunized mice were incubated sequentially for 30 min at 4°C with FITC-conjugated anti-mouse γδ TCR or αβ TCR or CD3 Abs and for 15 min at 4°C with anti-FITC Microbeads (Miltenyi Biotec GmbH, Bergisch Gladbach, Germany). The cells were then separated into bound and non-bound fractions on an autoMACS^TM^ separator column (Miltenyi Biotec GmbH). The purity of the isolated cells was >95%, as determined by flow cytometric analysis using PE-conjugated Abs against αβ or γδ T cells.

More than 60–80% of the γδ T cells isolated from immunized mice are already activated and express high levels of CD69, CD44 and IL-23R. The activation of γδ T cells was confirmed by the detection of IL-17 production and the staining of multiple activation markers, including CD69, CD44 and CD25.

### Generation of bone marrow dendritic cells

Bone marrow dendritic cells (BMDCs) were generated by incubation of bone marrow cells for 5 days in the presence of 10 ng/ml of recombinant murine GM-CSF and IL-4 (R&D Systems), as described previously [30].

### Assessment of the ex vivo effects of γδ T cell on Foxp3 T cell response

CD3^+^ T cells were isolated from IRBP_1-20_ immunized B6 or TCR-δ^-/-^ mice 13 days post-immunization, with or without a prior injection of γδ T cells. The percentage of Foxp3^+^ T cells among αβ T cells was determined by intracellular staining with PE-conjugated anti-Foxp3 Abs and APC-conjugated anti-mouse αβTCR Abs. T cells were cultured in a 24-well plate in the presence or absence of a low dose of recombinant IL-2 (1 ng/ml). Five days later, the percentage of Foxp3^+^ among the responder T cells was determined by intracellular staining. To further determine in vivo regulatory effect of γδ T cells on Foxp3 response, the Foxp3 responses were compared among immunized TCR-δ^-/-^ mice, with or without a prior injection of activated γδ T cells (1 x 10^6^/mouse), after a 5 day culture of the CD3^+^ responder T cells in low dose IL-2.

### Adenosine assay

Adenosine in the medium of cultured cells is measured by a multi-step enzymatic approach that results in the generation of an intermediate that reacts with the adenosine probe with the formation of a fluorescent product that can be detected by a fluorometric adenosine assay adenosine deaminase followed the fluorescent product is measured at Ex/Em = 535/587 nm. The result of reaction was read in a spectraMax iD5 multi-mode microplate reader (Molecular Devices,LLC. USA). The assay kit (Catalog # K327-100) was provided by Biovision (CA).

### Statistical analysis

The results in the figures are representative of one experiment, which was repeated 3–5 times. The statistical significance of differences between groups in a single experiment was initially analyzed by ANOVA; if statistical significance was detected, the Student–Newman–Keuls post-hoc test was subsequently used. P values less than 0.05 were considered a statistically significant difference and marked with one *; when P<0.01, two ** were used.

## Results

### Augmented Foxp3^+^ T cell response in γδ-deficient mice

**Fig 1:**
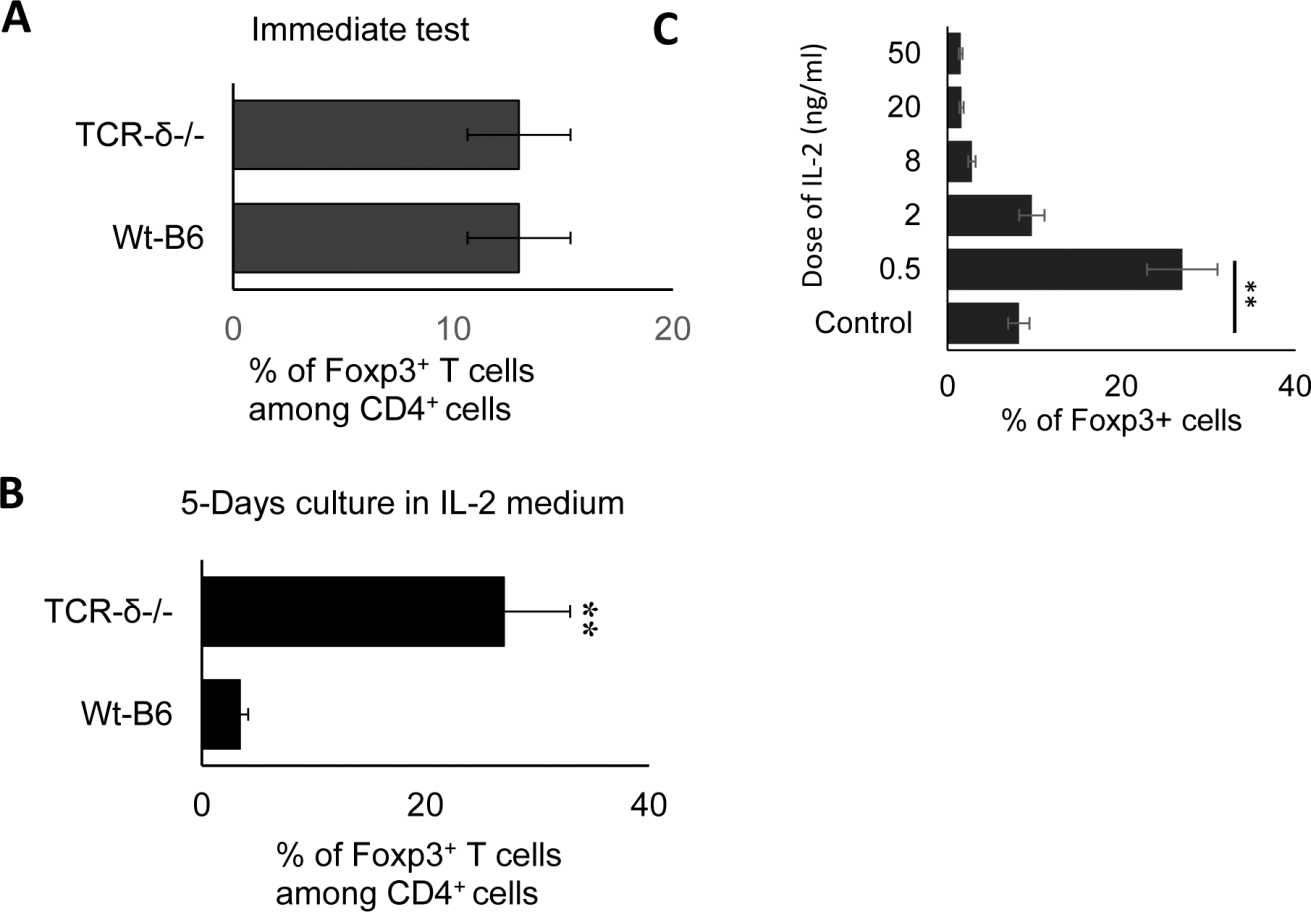
When cultured in medium containing low concentrations (1 ng/ml) of IL-2, a significantly large proportion of TCR-δ^-/-^ T cells, as compared to wt-B6 T cells, expressed Foxp3. A&B). Groups (n = 6) of B6 and TCR-δ^-/-^ mice were immunized with IRBP_1-20_/CFA. Thirteen days after immunization, CD3^+^ cells were separated from spleen and draining lymph nodes cells using a MACS column. They were stained with anti-mouse CD4 and anti-Foxp3 antibodies, either immediately (A) or after a 5-day culture in medium containing 1ng/ml IL-2 (B). Data are from one single experiment, which are representative of multiple (>5) independent experiments. C). Low dose of IL-2 favors Foxp3 T cell response. CD3^+^ cells separated from TCR-δ^-/-^ mice were cultured in medium containing indicated amounts of IL-2. Five days later, the proportion of Foxp3^+^ T cells was evaluated by FACS analysis, after staining with anti-Foxp3 antibodies.

To determine the cellular and molecular mechanisms by which activated γδ T cells modulate the Foxp3 T cell response, we compared the Foxp3 T cell response of B6 (wt control) and TCR-δ^-/-^ mice, which lack γδ T cells. CD3^+^ responder T cells were isolated from draining lymph nodes and spleens of immunized B6 and TCR-δ^-/-^ mice using a MACS column. Foxp3^+^ cells among the CD3^+^ cells were assessed by FACS analysis after antibody staining. The ex vivo relative frequency of Foxp3^+^ cells did not differ significantly between the two strains of mice (Fig 1A) but after a 5-day culture in medium containing a low dose of 1 ng/ml of IL-2, the frequency of these cells was much greater in the TCR-δ^-/-^ cultures (Fig 1B). The IL-2 concentration in the culture medium is critical in determining the relative frequency of Foxp3^+^ T cells in this experimental setting. While low concentrations of IL-2 (0.5–2 ng/ml) favor Foxp3 T cells, higher concentrations favor effector T cells (defined as Foxp3^-^ T cells) (Fig 1C).

### Activated γδ T cells efficiently inhibit the Foxp3^+^ T cell response

**Fig 2:**
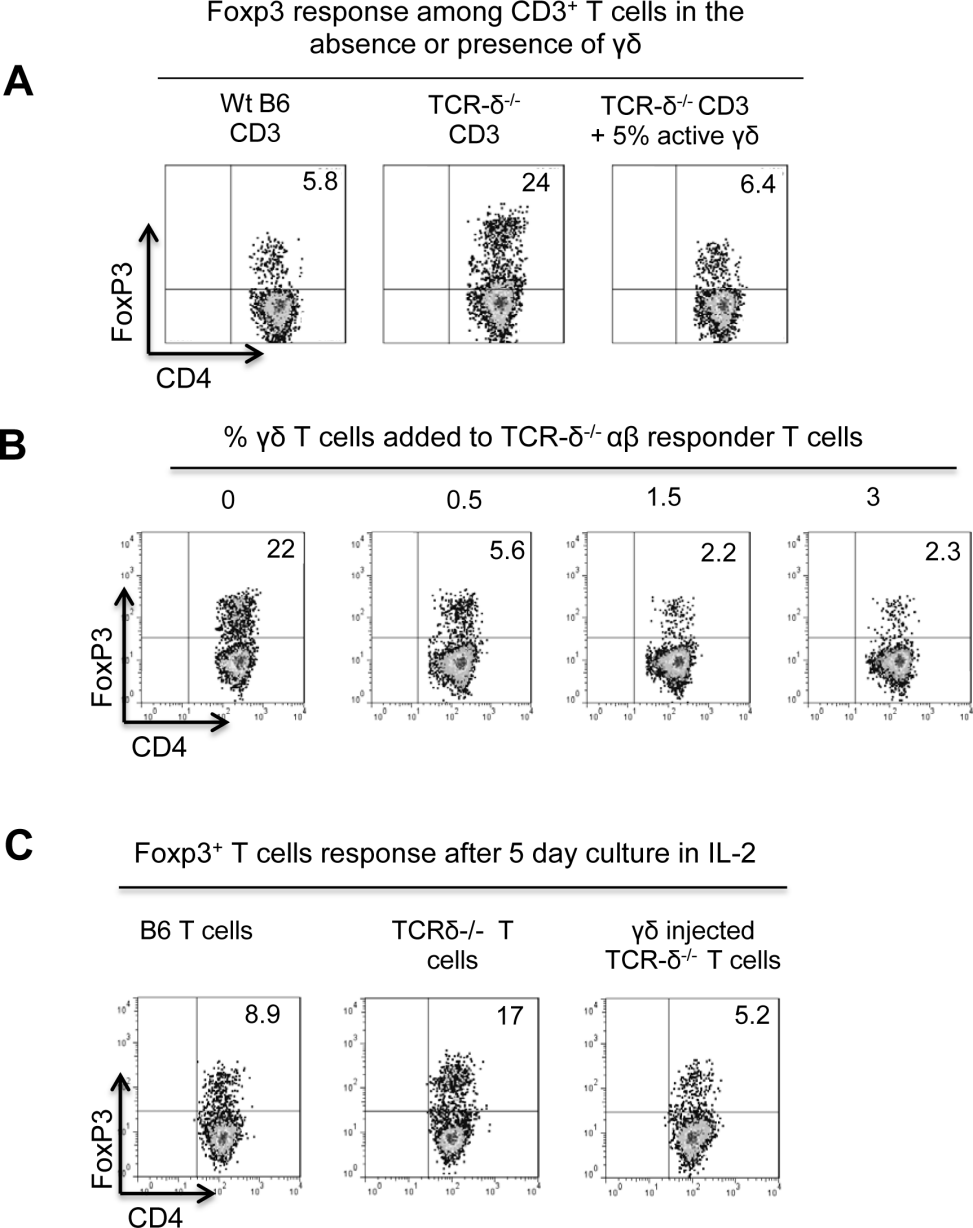
γδ T cells possess an inhibitory effect on the Foxp3 response. A). Addition of γδ T cells to TCR-δ^-/-^ responder T cells inhibited the Foxp3 T cell response. CD3^+^ T cells (1 x 10^6^/well) prepared from immunized TCR-δ^-/-^ mice were cultured in the presence or absence of isolated γδ T cells (5 x 10^4^/well) from immunized B6. After culture in medium containing 1ng/ml IL-2 for 5 days, the percentage of Foxp3^+^ T cells among CD3^+^ T cells was determined by FACS analysis. B). Addition of a minimum of 1% γδ T cells to TCR-δ^-/-^ responder T cells effectively inhibited the Foxp3 T cell response. TCR-δ^-/-^ responder T cells were cultured in medium containing 1 ng/ml recombinant IL-2 in the presence or absence of different numbers of isolated γδ T cells from immunized B6 mice. The percentage of Foxp3^+^ cells among the responder T cells were assessed at the end of five day cultures. C). Administration to TCR-δ^-/-^ mice with 1 x 10^6^/mouse of γδ T cells inhibited the Foxp3 T cell response. TCR-δ^-/-^ mice were injected with 1 x 10^6^/mouse γδ T cells, isolated from immunized B6 mice, before they were immunized. Thirteen days after immunization, Foxp3^+^ T cells were evaluated. Data are from one single experiment and are representative of multiple (>5) independent experiments.

To avoid γδ interference on the Foxp3 T cell response, αβ T cells were prepared from immunized TCR-δ^-/-^ mice and the Foxp3 response was determined with or without added γδ T cells. We found that under these conditions Foxp3^+^ T cells were elevated approximately 4-fold among TCR-δ^-/-^ CD3^+^ cells as compared to B6 CD3^+^ cells. The addition of a small number γδ T cells to TCR-δ^-/-^ cultures reduced Foxp3^+^ T cells to wt levels (Fig 2A). Furthermore, titrating the inhibitory effect of γδ T cells revealed that as few as 0.5% γδ T cells effectively reduced the expansion of Foxp3^+^ cells, and the maximal inhibition was reached at or below 3% of γδ T cells (Fig 2B).

To determine if γδ T cells inhibit the Foxp3 T cell response in vivo as well, two groups (n = 8) of TCR-δ^-/-^ mice were immunized with the uveitogenic peptide IRBP_1-20_/CFA. One group was injected with activated γδ T cells (1 x 10^6^/mouse) before the immunization. The frequency of Foxp3^+^ cells among CD3^+^ T cells at 13 days post-immunization was determined. By comparison with the non-injected TCR-δ^-/-^ mice, the γδ T cell-injected mice showed a significantly decreased frequency of Foxp3^+^ T cells (Fig 2C).

**Fig 3:**
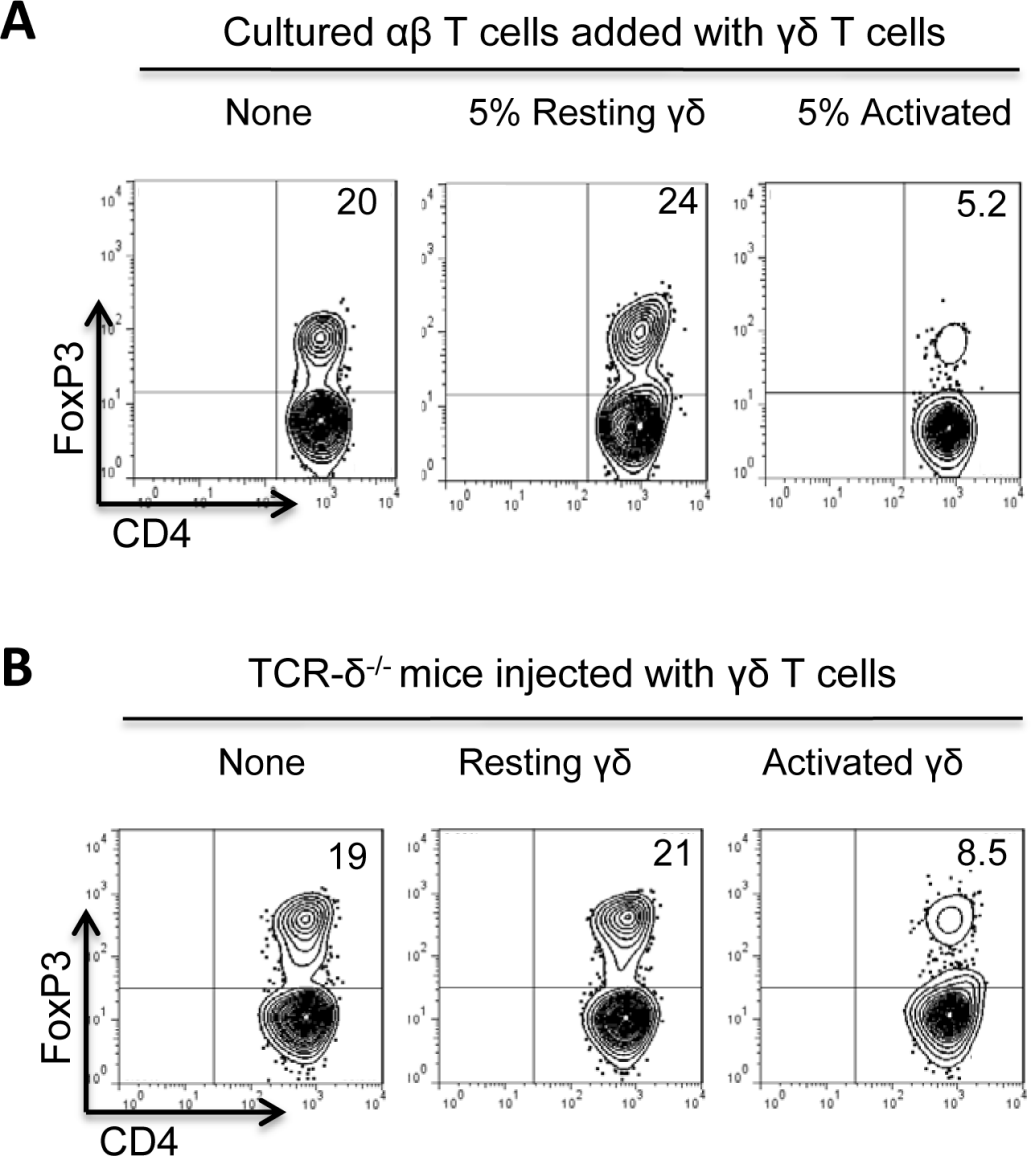
Enhancing effect on Foxp3 T cell response is a feature of activated, but not non-activated, γδ T cells. A) Non-activated and activated γδ T cells were isolated from naïve or immunized B6 mice, respectively [14,18]. CD3^+^ T cells isolated from immunized TCR-δ^-/-^ mice were cultured with non-activated and activated γδ T cells (1 x 10^6^/mouse). After 5 days, Foxp3 T cell responses were evaluated as Fig 2A. B) Administration to TCR-δ^-/-^ mouse of activated, but not non-activated, γδ T cells inhibited Foxp3 T cell response in vivo. Administration of a small number (1 x 10^6^/mouse) of activated, or non-activated γδ T cells into TCR-δ^-/-^ mouse before immunization. After thirteen days, Foxp3^+^ T cells were evaluated as Fig 2C.

Our previous studies demonstrated that activated γδ T cells are functionally different from resting γδ T cells [7,14,18]. To compare their differential effect on Foxp3^+^ T cells, either resting or activated γδ T cells were added to αβ responder T cells at a ratio of 1:20 (5%) before culturing in IL-2^low^-containing medium. Activated γδ T cells suppressed the Foxp3 T cell response, whereas resting γδ T cells did not (Fig 3A). Likewise, injection of a small number of activated, but not resting, γδ T cells (1 x 10^6^/mouse) to a TCR-δ^-/-^ mouse before immunization significantly inhibited the Foxp3 T cell response in vivo (Fig 3B).

### Supernatant of γδ T cells enhances the Foxp3 T cell response

**Fig 4:**
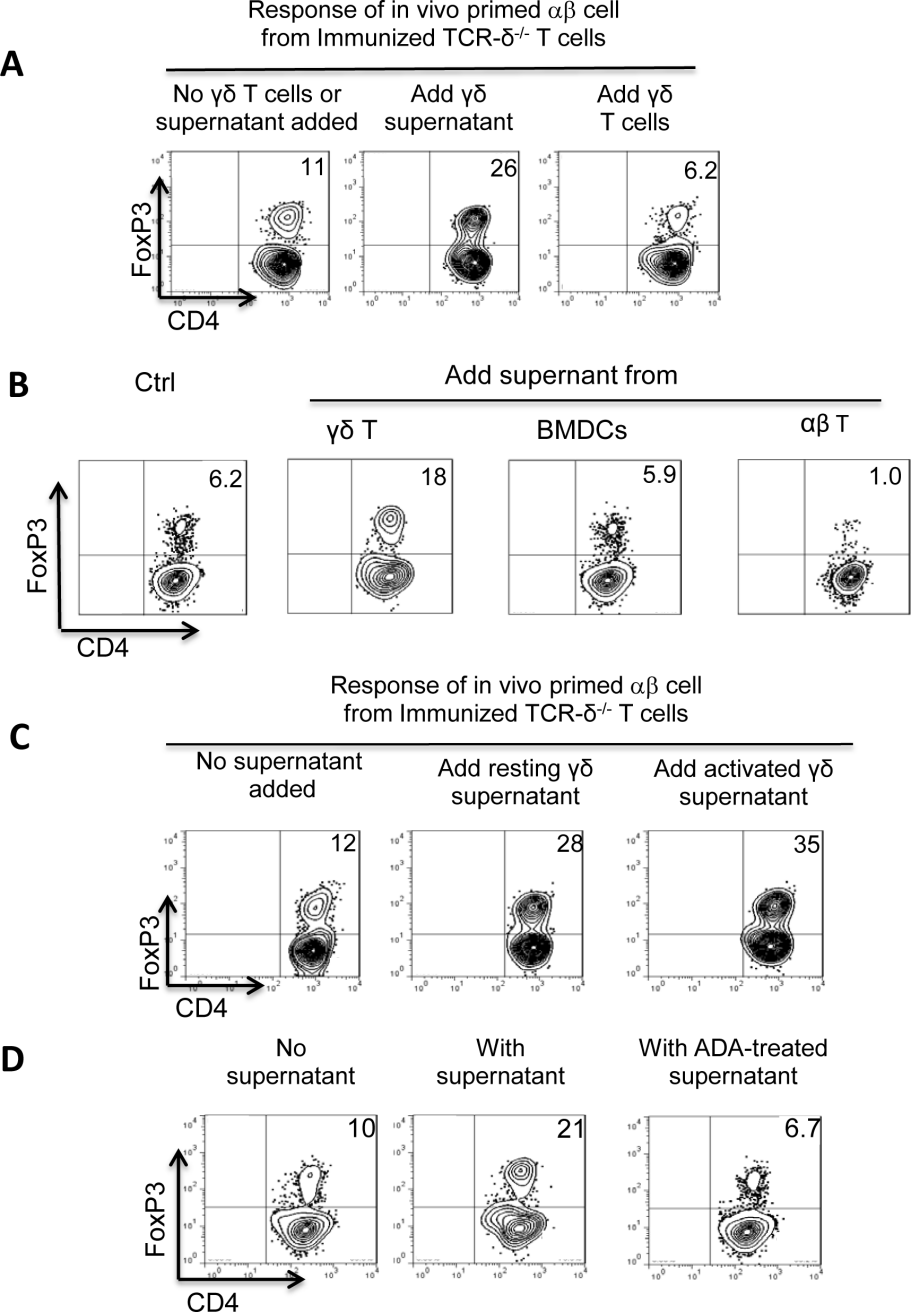
Supernatants of γδ T cells enhanced the Foxp3 T cell response. TCRαβ and CD3^+^ T cells were purified from B6 or TCR-δ^-/-^ mice immunized with the human interphotoreceptor retinoid-binding protein (IRBP) peptide IRBP_1-20_, as described previously; non-activated and activated γδ T cells were prepared by isolation from naïve and immunized mice, respectively (materials and methods). Two-day culture supernatants were collected from γδ T cells (at a density of 1 x 10^6^/well of 24-well plate). They were tested by addition to TCR-δ^-/-^ responder T cells at a volume of 1:5. A) Addition of γδ T cell supernatant enhanced, whereas direct addition of γδ T cells into the culture inhibited the Foxp3 T cell response. In a 24-well plate, 1 x 10^6^/well TCR-δ^-/-^ responder T cells were cultured in IL-2-containing medium in the absence or presence of activated γδ T cells (2%, right panel) or γδ T cell supernatant (1:5 vol:vol, middle panel). Foxp3^+^ T cells were evaluated as detailed before. B) Supernatants of cultured γδ, but not dendritic cells and αβ T cells, enhanced the Foxp3 T cell response in vitro. Supernatants collected from γδ T cells, BMDCs, and αβ T cells (at a density of 1 x 10^6^/well of 24-well plate) were added to TCR-δ^-/-^ responder T cells at a volume of 1:5. Foxp3^+^ T cells were evaluated as detailed before. C) The culture supernatants of both activated and non-activated γδ T cells had an enhancing effect on Foxp3 T cell response in culture. Supernatants collected from non-activated γδ T cells from naïve mice and activated γδ T cells from immunized mice [15,18,29] were added to TCR-δ^-/-^ responder T cells at volume of 1:5. Foxp3^+^ T cells were evaluated as detailed before. D) The enhancing effect of the γδ T cell supernatants was abolished after treatment by adenosine-degrading enzyme (ADA). γδ supernatants, with or without a prior treatment of ADA (1U/ml), were added to TCR-δ^-/-^ responder T cells at volume of 1:5,. Foxp3^+^ T cells were evaluated as detailed before.

To determine whether direct cell-cell contact is required for the inhibitory γδ-Foxp3 T cell interaction, 48h cultured γδ T cell supernatants were collected and added to αβ responder T cells, at a v/v ratio of 1:5. Unexpectedly, the γδ T cell supernatant enhanced the Foxp3 T cell response, whereas addition of γδ T cells inhibited the response (Fig 4A). Subsequent experiments showed that supernatants collected from cultures of dendritic cells (DCs) were not enhancing, and those from αβTCR^+^ T cells were actually inhibitory (Fig 4B).

Meanwhile, supernatants collected from activated and non-activated γδ T cells had a similarly enhancing effect (Fig 4C). We also tested the effect of γδ T cell supernatants after incubation with an adenosine-degrading enzyme ADA (Fig 4D) [31–33]. Interestingly, after ADA treatment the γδ T cell supernatant lost its enhancing effect (Fig 4D, right panel).

**Fig 5:**
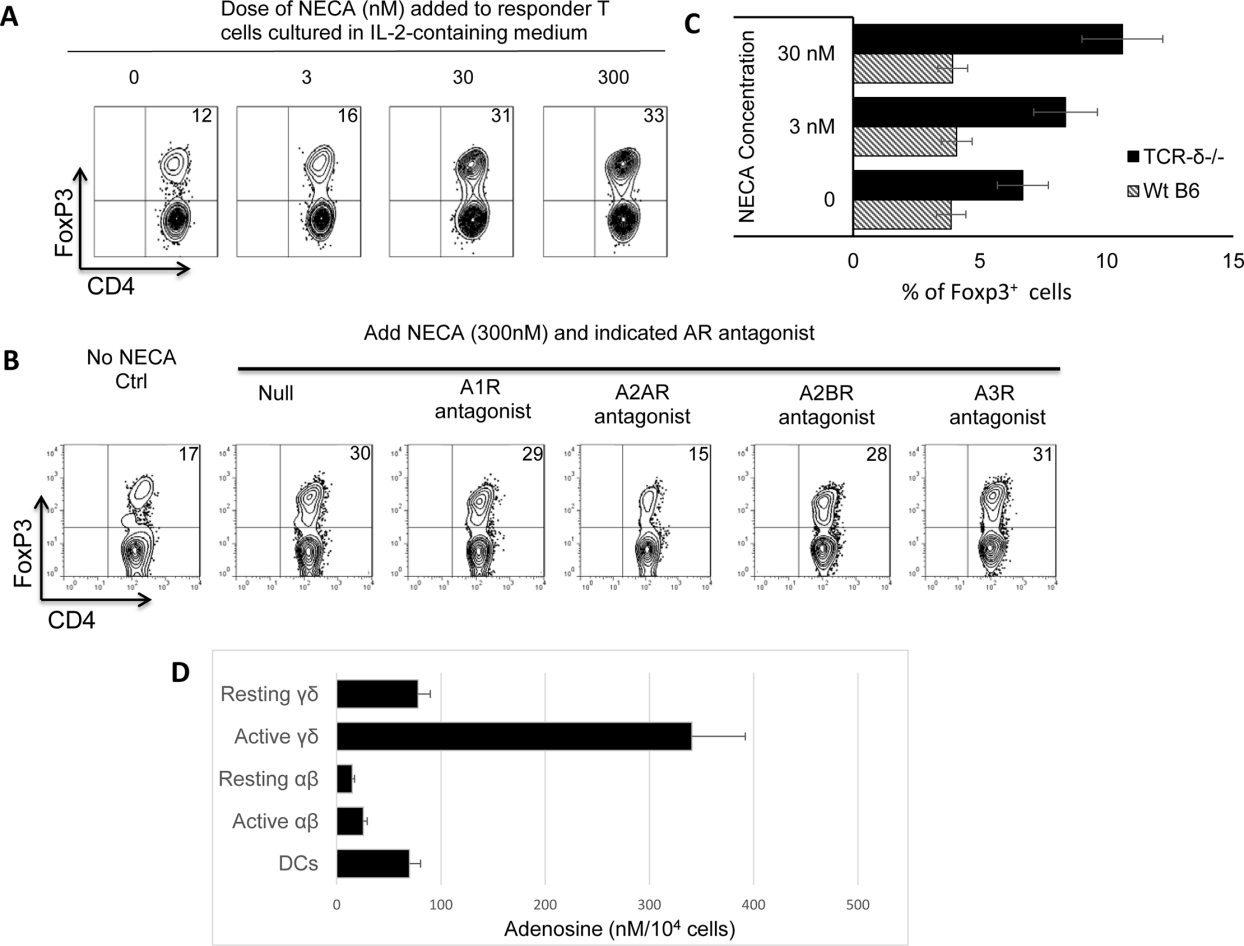
Adenosine analogue (NECA) enhanced Foxp3 T cell response at very low doses. A) NECA (3–30 nM) significantly enhanced Foxp3 T cell response. Titrated doses of NECA were added to TCR-δ^-/-^ responder T cells cultured in 1 ng/ml of recombinant IL-2. Five days later the Foxp3^+^ cells were assessed as described earlier. B) The enhancing effect of NECA was offset by A2AR antagonist, but not by antagonists specific for other adenosine receptors (A1R, A2BR, and A3R). TCR-δ^-/-^ responder T cells were cultured in medium containing 1 ng/ml of recombinant IL-2 and 300 nM NECA, in the presence of indicated adenosine receptor antagonists: A1R antagonist (DPCPX, 50nM); A2AR antagonist (SCH58261, 100nM); A2BR antagonist (MRS1754, 100nM); and A3R antagonist (MRS 1220, 5 μM). Five days later the Foxp3^+^ cells were assessed as described earlier. C) Adenosine-mediated enhancing effect on Foxp3 T cell response is regulated by γδ T cells. Responder T cells isolated from immunized wt-B6 and TCR-δ^-/-^ mice, 13 days post-immunization were cultured in medium containing 1 ng/ml of IL-2 and indicated doses of NECA. Five days later the Foxp3^+^ cells were assessed as described earlier. D) Supernatants of cultured γδ T cells contained higher amounts of adenosine. 25 μl of supernatant samples of indicated immune cells (αβ/γδ T cells and DCs) were mixed with assay buffer, adenosine convertor, adenosine detector, adenosine developer and adenosine probe in room temperature and protected from exposure to light for 15 minute. Fluorescence amounts generated as a result of reaction was read in a spectraMax iD5 multi-mode microplate reader (Molecular Devices,LLC. USA) at Ex/Em = 535/587 nm.

To determine whether adenosine is the effector molecule in the supernatant of cultured γδ T cells, we tested the effect of the adenosine analogue (NECA) on the Foxp3 response. Fig 5 shows that adding NECA at low concentrations (3–30 nM) significantly enhanced the Foxp3 response (Fig 5A). Moreover, a co-added selective A2AR antagonist abolished this enhancing effect, whereas antagonists specific for other adenosine receptors (A1R, A2BR, and A3R) did not (Fig 5B). Finally, the NECA effect on the Foxp3 response was only evident with TCR-δ^-/-^ responder cells (Fig 5C), suggesting that the endogenous γδT cells in the B6 T cell cultures interfere with the adenosine-driven Foxp3 T cell response.

To further quantify the adenosine generated in the supernatant of cultured γδ T cells, we have performed a multi-step enzymatic approach that directly assessed the adenosine amounts in the supernatants of cultured αβ/γδ T cells and DCs (Fig 5D). The results showed that both non-activated and activated γδ T cells generate a lot more adenosine as compared to αβ T cells and DCs.

### A2AR^-/-^ γδ T cells are unable to suppress the Foxp3^+^ T cell response

**Fig 6:**
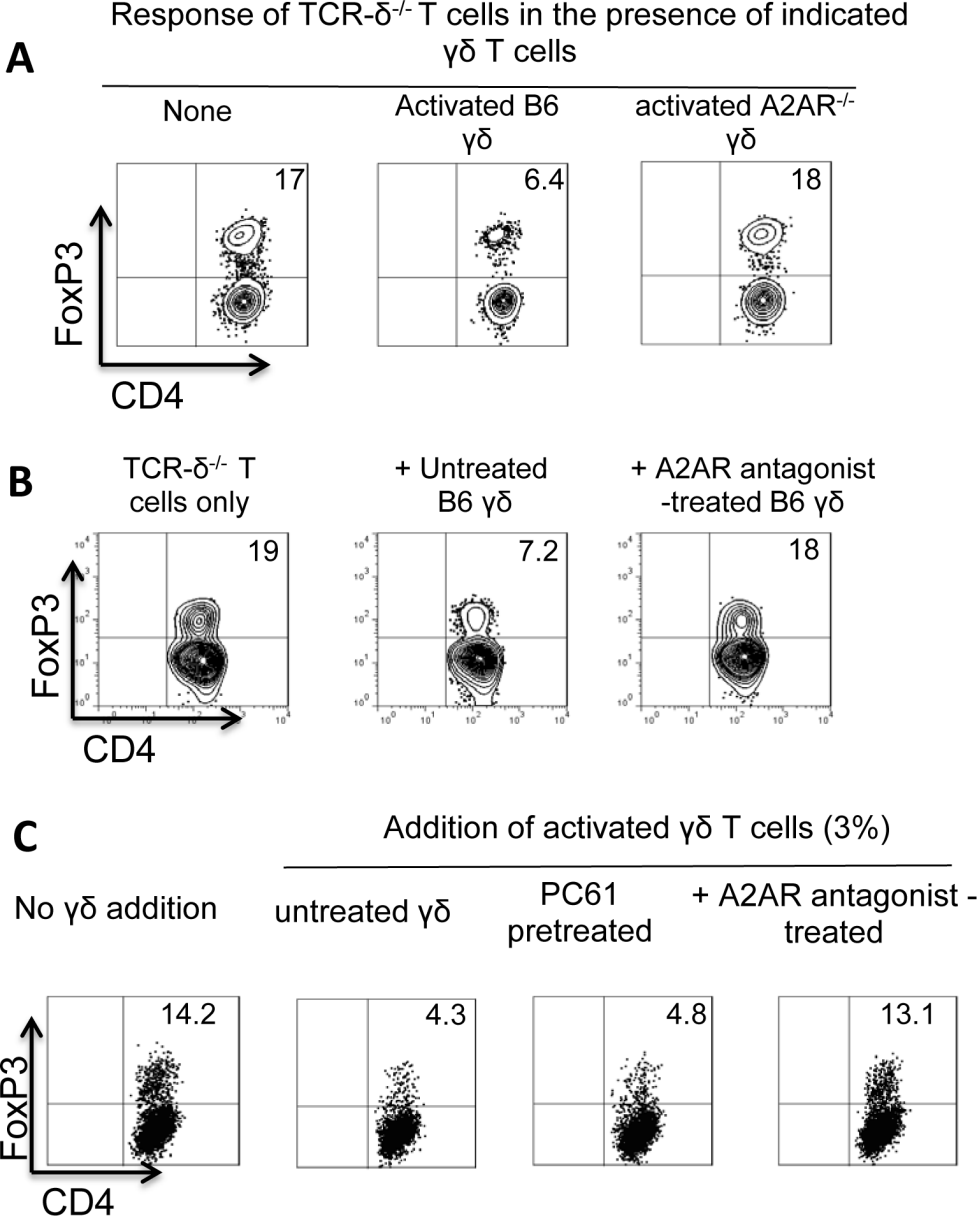
A2AR^-/-^ γδ T cells were unable to suppress Foxp3^+^ T cell response. A) CD3^+^ responder T cells isolated from immunized TCR-δ^-/-^ mice were cultured with 1 ng/ml recombinant IL-2 in the presence or absence of γδ T cells isolated from immunized B6 or A2AR^-/-^ mice and subsequently stained with a PE-labeled anti-Foxp3 antibody. B) Pretreatment of A2AR^+/+^ γδ T cells with an A2AR antagonist (SCH 58261) abolished the enhancing effects. CD3^+^ responder T cells isolated from immunized TCR-δ^-/-^ mice were cultured with 1 ng/ml recombinant IL-2 for 5 days, in the absence (left panel) or presence (middle and right panels) of γδ T cells that were treated (right panel) or untreated with an A2AR antagonist. Foxp3^+^ T cells in the cultures were identified. C) Blockade of high-affinity IL-2 receptor CD25 by monoclonal antibody (PC61) did not affect the inhibiting effect of γδ T cells. CD3^+^ responder T cells isolated from immunized TCR-δ^-/-^ mice were cultured with 1 ng/ml recombinant IL-2 for 5 days, in the absence (left panel) or presence (three right panels) of activated γδ T cells that were treated as indicated.

Because activated γδ T cells were better inhibitors of the Foxp3^+^ T cell response studied here, and because we previously observed that activated γδ T cells expressed increased amounts of A2AR [15,18], we wished to determine whether altered expression A2AR contributes to the functional change. To test this, we compared the suppressive effect of A2AR^-/-^ and A2AR^+/+^ γδ T cells. We found that A2AR^-/-^ γδ T cells were completely deficient in this suppressive activity (Fig 6A).

We also tested whether treatment of A2AR^+/+^ γδ T cells with an A2AR antagonist (SCH 58261) would affect the inhibitory activity. To this end, activated A2AR^+/+^ γδ T cells isolated from immunized B6 mice were added to responder αβ T cells prepared from immunized TCR-δ^-/-^ mice, with or without pre-incubation with the A2AR antagonist. We found that pre-treatment of A2AR^+/+^ γδ T cells with the A2AR-specific antagonist abolished their Foxp3-inhibitory effect (Fig 6B). We have now conducted experiment, in which the effect of γδ T cells on Foxp3 responses was tested with or without a prior incubation with anti-IL-2R antibody (PC61). Our results showed that blockade of high-affinity IL-2 receptor CD25 does not affect the inhibiting effect of γδ T cells (Fig 6C), suggesting that the possibility of competition for IL-2 between Tregs and γδ T cells can be ruled out.

**Fig 7:**
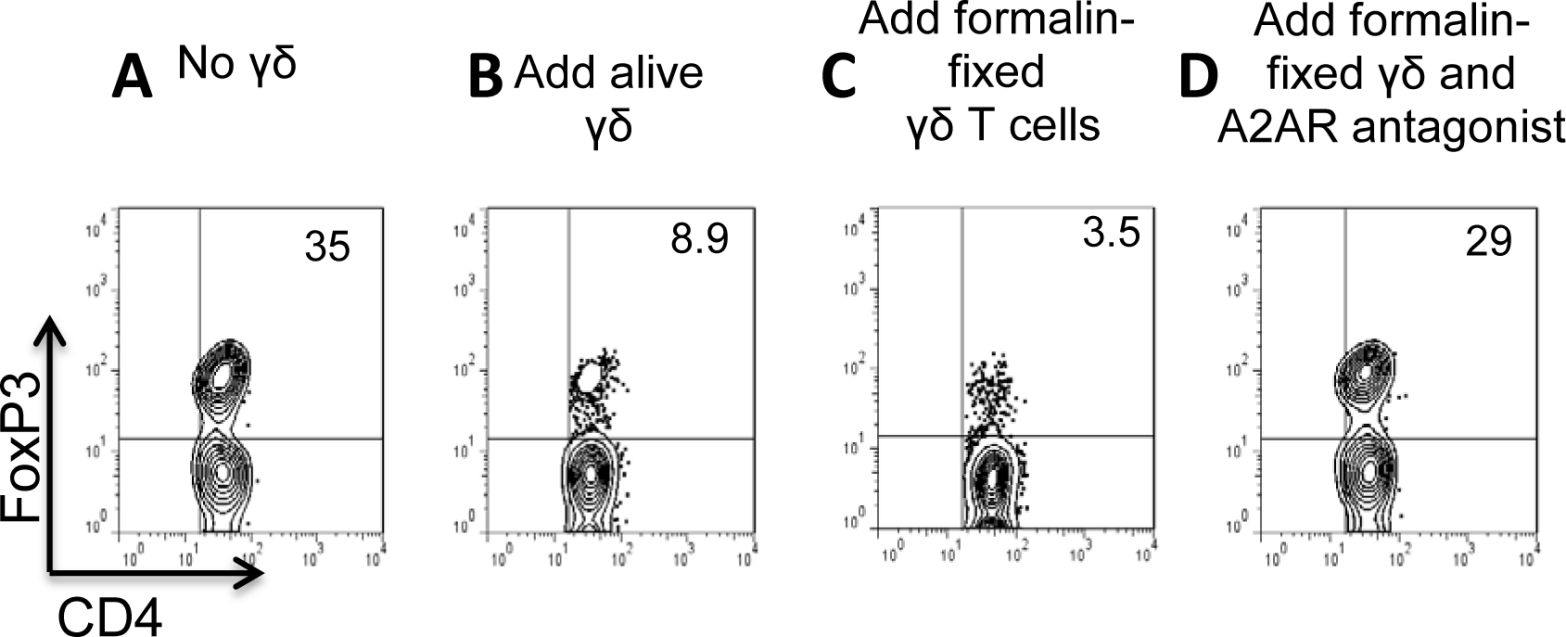
The formalin-fixed γδ T cells were as effective as live γδ T cells in inhibition of Foxp3 T cell response. In a 24-well plate, αβ responder T cells (1 x 10^6^/well] isolated from immunized TCR-δ^-/-^ mice were cultured in IL-2-containing medium (1 ng/ml) for 5 days with (7B-7D) or without (7A) addition of γδ T cells–either alive (7B) or formalin-fixed (7C&D) and in the absence (Fig 7A–7C) or presence (Fig 7D) of an A2AR antagonist (SCH58261, 100nM). Results shown are from a single experiment and are representative of three experiments.

We previously demonstrated that formalin-fixed γδ T cells retained the ability to bind adenosine [34]. We decided to test whether formalin-fixed γδ T cells retain their inhibitory effect on the Foxp3 T cell response. As shown in Fig 7, formalin-fixed γδ T cells (7C) were as effective as alive cells in inhibition (Fig 7B). Moreover, as with live γδ T cells, the inhibitory activity was almost completely abolished by the addition of an A2AR antagonist (Fig 7D).

## Discussion

Although the regulatory effect of γδ T cells on inflammation and immune responses has been shown repeatedly [3,4,6,12,35,36], the mechanism by which they regulate immunity remains largely unknown. Working in a reproducible EAU model, we have previously shown that γδ T cells effectively regulated the pathogenesis of EAU; that γδ T cells could either inhibit or enhance the autoimmune response in EAU [6,18]; that activated γδ T cells possessed an increased enhancing effect on autoimmune responses, whereas non-activated γδ T cells are less effective [7,14,18]. We also reported that the regulatory effect of γδ T cells is not a stable feature; instead, the enhancing and inhibiting effect changes with γδ T cell activation status [4,7,37]. In the current study we demonstrate that the functional conversion of γδ T cells is closely associated with their effect on the Foxp3 T cell response.

Our assay system for detecting the Foxp3 T cell response consists of a 5-day culture of responder T cells in medium containing low doses (1–2 ng/ml) of IL-2. Such conditions promoted the response of regulatory T cells, in agreement with previous reports that a low dose of IL-2 favors regulatory T cell function [15,38], whereas high doses favor responses of effector autoreactive T cells [38,39].

The regulatory effect of γδ T cells on the Foxp3 T cell response has been previously described [2,4,40,41]. While some studies reported that γδ T cells have an inhibitory effect [4,40], the opposite effect was also seen [2,38,41]. For example, it was reported that γδ T cells can antagonize regulatory T cell-mediated suppression of αβ T cells and that soluble mediators produced from IL-23-activated γδ T cells are involved in this process [4]. There are also studies showing that the proportion of in vivo-generated regulatory T cells was greatly enhanced in mice deficient in γδ T cells (29). But how γδ T cells control regulatory T cells remained largely unclear.

In this study, we observed that γδ T cells can either increase or diminish regulatory T cell populations. Cell-free supernatants of γδ T cells had an enhancing effect on the Foxp3 T cell response, whereas γδ T cells themselves, particularly in an activated state, strongly inhibited the Foxp3 T cell response. These effects reflected a balance of adenosine production and absorption by γδ T cells, which determined the net effect on Foxp3^+^ regulatory T cells.

The effect of adenosine on the regulatory T cell function has been reported in previous studies [38,42–45]; for example, an adenosine analogue enhanced the Foxp3 response [15,38] while inhibiting the non-Foxp3 T cell response [38,39]. They also showed that the development of immunosuppressive Foxp3^+^ T cells was influenced by the adenosine-A2A adenosine receptor pathway [46]. Adenosine is an extracellular purine nucleoside signaling molecule, which governs cell and tissue function in both health and disease. Adenosine is formed after the degradation of its precursor, adenosine 5′-triphosphate (ATP), a process which can take place both extra- and intracellularly. ATP, a predominantly intracellular molecule, is released from stressed cells and is degraded to adenosine through a cascade of ectonucleotidases, including CD39 (nucleoside triphosphate diphosphorylase [NTPDase]) and CD73 (5′-ectonucleotidase [Ecto5′NTase]) [47,48]. In inflammation, multiple cell types release ATP or ADP from their intracellular compartments into the extracellular space and results in accumulation of adenosine [49].

We have shown in this study that the Foxp3 T cell response is significantly inhibited in the presence of activated γδ T cells, consistent with our previous observation that activated γδ T cells can enhance autoimmune responses [7,8,14,18]. However, we also observed that the inhibiting effect of γδ T cells can be completely abolished by blocking their surface A2AR adenosine receptor; that γδ T cell supernatants, regardless of the activation status of the cells, have an enhancing effect on the Foxp3 T cell response; that the enhancing effect in γδ T cell supernatants is significantly diminished after pre-treatment by ADA, an adenosine-degrading enzyme. Thus, γδ T cells constantly enhance the Foxp3 T cell response, with the inhibitory effect predominant only when γδ T cells are activated [4,40].

In conclusion, modulation of the Foxp3 T cell response by γδ T cells is dependent on the production of adenosine by γδ T cells and the ability of γδ T cells to bind and absorb the molecule. When γδ T cells are activated and their adenosine-binding activity is greatly increased there is a net-decrease in extracellular adenosine resulting in inhibition of Foxp3^+^ T cells and enhancement of autoimmunity.

## Supporting information

S1 File The ARRIVE guidelines checklist. (PDF)
